# Supplementary material for: The Health Behaviour of German Outpatient Caregivers in Relation to the COVID-19 Pandemic: A Mixed-Methods Study
Source: Int J Environ Res Public Health. 2021 Aug 3;18(15):8213. doi: 10.3390/ijerph18158213 (PMC8346166; doi:10.3390/ijerph18158213)
Supplement: Supplementary file 1 [file ijerph-18-08213-s001.zip › ijerph-1276451-supplementary.pdf]

International Journal of
Environmental Research and Public Health

MDPI

Supplementary Material

Table S1. Interview topic list.

Phase of the interview	Contents
1 Information phase	Introduction: study information, confidentiality, informed consent
2 Warm-up phase	Qualifications, working activity
3 Main phase	Health behaviour, health-promoting behaviour during the COVID-19 pandemic
4 Final phase and end of the interview	Socio-demographics of the interviewees and fare-well

Table S2. Variables of the study instrument used in the analyses.

Variables.	Name and source of the original scales and items	Items (*n*)
Socio-demographic factors		20
Health behaviour		
Eating behaviour	Mediterranean Dietary Assessment Score (MEDAS): validated German version	14
Physical Activity	Global Physical Activity Questionnaire (GPAQ): validated German version	16
Smoking	Smoking exposition questionnaire	7
Sleep quality	Pittsburgh-Sleep-Quality-Index (PSQI): single-item	1

Table S3. Self-administered variables of the study instrument used in the analyses.

Variables	Self-developed questions	Items (n)
**Health behaviour**		
Drinking behaviour	• *How much do you drink in total per day?*<br>• *Do you drink beverages containing caffeine, such as coffee or coffee specialties, coke or energy drinks?* - Yes / No<br>• *If yes, how many cups (200ml) of coffee or coffee specialties and glasses of coke or energy drinks do you drink a day?*<br>• *Does your work influence a decreased drinking behaviour?* - Yes / No<br>• *If yes, what are the causes of your reduced drinking behaviour during working hours?* - I do not have the time / there are no toilets available / I don't want to go to the toilet with the carers / I forget to drink / I forget to bring something to drink with me / other reasons	5
Health behaviour change since pandemic	• *How would you rate your eating behaviour since the beginning of the COVID-19 pandemic?* - I currently eat much unhealthier [-2] / unhealthier [-1] / just as healthy [0] / healthier [1] / much healthier [2] than before.<br>• *How would you rate your physical activity since the beginning of the COVID-19 pandemic?* I am currently much less [-2] / less [-1] / just as [0] / more [1] / much more [2] physically active than before.<br>• *How would you rate your smoking habits since the beginning of the COVID-19 pandemic?* - I currently smoke a lot less [-2] / less [-1] / as much as [0] / more [1] / a lot more [2] than before.<br>• *Since the beginning of the COVID-19 pandemic, my life has become more stressful than before!* - I do not agree at all [-2] / I do not agree. [-1] / I neither agree nor disagree. [0] / I agree. [1] / I totally agree. [2]<br>• *How would you rate your sleep behaviour since the beginning of the COVID-19 pandemic?* - I currently sleep much worse [-2] / worse [-1] / neither worse nor better [0] / better [1] / much better [2] than before.	5

Table S4. Sub-group analysis of health behaviours and self-perceived health behaviour change since COVID-19 pandemic for gender, age and BMI groups.

Outcome	**M (SD)** **Male** **(*n* = 57)** [b]	**M (SD)** **Female** **(*n* = 112)** [b]	**z** [a]	***p***	**M (SD)** **<40 years** **(*n* = 73)**	**M (SD)** **≥40 years** **(*n* = 98)**	**z** [a]	***p***	**M (SD)** **Normal weight** [c] **(*n* = 101)**	**M (SD)** **Overweight** [c] **(*n* = 70)**	**z** [a]	***p***
Dietary Quality Score [d]	5.4 (±2.04)	6.7 (±2.22)	**4342**	**<0.001**	6.1 (±2.29)	6.4 (±2.18)	3875	0.348	6.6 (±2.03)	5.9 (±2.43)	**2795**	**0.019**
Physical Activity [d,e]	8919.2 (±7875.45)	10268.9 (±7871.47)	3116.5	0.219	9432.5 (±7480.50)	10070.1 (±8111.87)	3297	0.674	9978.6 (±8232.92)	9521.4 (±7231.08)	3066.5	0.897
Tobacco consumption [d,f]	19.4 (±22.15)	18.3 (±16.51)	999.5	0.621	11.2 (±13.09)	22.9 (±19.87)	**1297.5**	**0.002**	18.9 (±17.56)	19.0 (±20.28)	983	0.847
Since COVID-19 pandemic, self-perceived change in…												
Eating behaviour	–0.39 (±0.92)	–0.07 (±0.78)	**3852**	**0.011**	–0.51 (±0.75)	0.05 (±0.84)	**4693**	**<0.001**	–0.11 (±0.69)	–0.30 (±1.03)	3058	0.085
Physical activity	–0.39 (±0.86)	–0.37 (±0.89)	3219.5	0.922	-0.47 (±0.94)	–0.30 (±0.82)	4005	0.150	–0.27 (±0.84)	–0.51 (±0.91)	3022	0.082
Smoking behaviour	–0.05 (±0.74)	0.03 (±0.73)	376.5	0.477	0.24 (±0.66)	–0.08 (±0.75)	264	0.160	–0.13 (±0.71)	0.22 (±0.74)	**465**	**0.030**
Perceived stress	0.07 (±1.27)	0.58 (±1.17)	**3923**	**0.012**	0.49 (±1.27)	0.35 (±1.18)	3288.5	0.354	0.27 (±1.24)	0.61 (±1.16)	4105	0.066
Sleep quality	–0.67 (±0.76)	–0.63 (±0.82)	3258.5	0.808	–0.77 (±0.83)	–0.56 (±0.77)	4008	0.139	–0.68 (±0.82)	–0.60 (±.77)	3600.5	0.821

Note. N = 171. [a] Mann-Whitney U test. [b] Two diverse participants were excluded from the sub-group analysis of gender (n = 169) as a group of 2 cannot be statistically compared. [c] Normal weight: 18.5 kg/m^2 < BMI < 25 kg/m^2. Overweight: BMI ≥ 25 kg/m^2. No participants were underweight. [d] Outcome variables: MEDAS as Dietary Quality Score, MET (= metabolic equivalent) for Physical Activity, Pack years for Tobacco Consumption. [e] Differing sample and sub-group sizes due to excluded participants; sex: n = 159, n_m = 52, n_f = 107; age: n_{age} = 161, $n_{<40}$ = 69, $n_{\geq 40}$ = 92; weight status: n_{weight} = 161, n_N = 97, n_O = 64. [f] Differing sample and sub-group sizes as pack years cannot be calculated for non-smokers; sex: n = 90, n_m = 33, n_f = 57; age: n_{age} = 91, $n_{<40}$ = 31, $n_{\geq 40}$ = 60; weight status: n_{weight} = 91, n_N = 53, n_O = 38. $p < 0.05$ for values in bold.

Table S5. Sub-group analysis of health behaviours for gender, age and BMI groups.

Items	*n* (%) Male [a] (*n* = 57)	*n* (%) Female [a] (*n* = 112)	Chi² (df)	*p*	*n* (%) <40 years (*n* = 73)	*n* (%) ≥40 years (*n* = 98)	Chi² (df)	*p*	*n* (%) Normal weight [b] (*n* = 101)	*n* (%) Overweight [b] (*n* = 70)	Chi² (df)	*p*
**Eating behaviour**												
Fruit and vegetables/day			1.76 (1) [c]	0.184			2.91 (1) [c]	0.088			0.87 (1) [c]	0.351
<5 pc.	50 (87.7)	89 (79.5)			56 (76.7)	85 (86.7)			81 (80.2)	60 (85.7)		
≥5 pc.	7 (12.3)	23 (20.5)			17 (23.3)	13 (13.3)			20 (19.8)	10 (14.3)		
Sweets, cookies/week			0.57 (1) [c]	0.451			0.05 (1) [c]	0.822			0.18 (1) [c]	0.676
<3 portions	24 (42.1)	54 (48.2)			33 (45.2)	46 (46.9)			48 (47.5)	31 (44.3)		
≥3 portions	33 (57.9)	58 (51.8)			40 (54.8)	52 (53.1)			53 (52.5)	39 (55.7)		
**Drinking behaviour**												
Caffeinated drinks/day			0.73 (2) [c]	0.695			1.67 (2) [c]	0.434			4.67 (2) [c]	0.097
No caffeine	11 (19.3)	16 (14.3)			14 (19.2)	13 (13.3)			21 (20.8)	6 (8.6)		
<5 cups	20 (35.1)	43 (38.4)			24 (32.9)	40 (40.8)			36 (35.6)	28 (40.0)		
≥5 cups	26 (45.6)	53 (47.3)			35 (47.9)	45 (45.9)			44 (43.6)	36 (51.4)		
Work influence on drinking behaviour			**12.19** [d]	**0.014**			1.82 (4) [c]	0.768			**11.94** [d]	**0.017**
No	21 (36.8)	23 (20.5)			20 (27.4)	24 (24.5)			34 (33.7)	10 (14.3)		
Rarely	11 (19.3)	16 (14.3)			14 (19.2)	14 (14.3)			17 (16.8)	11 (15.7)		
Some	6 (10.5)	33 (29.5)			15 (20.5)	25 (25.5)			24 (23.8)	16 (22.9)		
High	17 (29.8)	30 (26.8)			18 (24.7)	29 (29.6)			20 (19.8)	27 (38.6)		
Very high	2 (3.5)	10 (8.9)			6 (8.2)	6 (6.1)			6 (5.9)	6 (8.6)		
**Physical activity** [e]												
Met WHO recommendation			1.90 [d]	0.616			1.96 [d]	0.621			6.15 [d]	0.084
Never meet	1 (1.9)	2 (1.9)			0 (0.0)	3 (3.3)			0 (0.0)	3 (4.7)		
150 min PA total	20 (38.5)	36 (33.6)			25 (36.2)	32 (34.8)			33 (34.0)	24 (37.5)		
150 min PA in recreational time	31 (59.6)	69 (64.5)			44 (63.8)	57 (62.0)			64 (66.0)	37 (57.8)		
**Smoking behaviour**												
Smoking status			1.04 (2) [c]	0.594			6.48 (2) [c]	0.039			0.06 (2) [c]	0.973
Non-smoker	24 (42.1)	55 (49.1)			42 (57.5)	38 (38.8)			48 (47.5)	32 (45.7)		
Ex-smoker	12 (21.1)	24 (21.4)			14 (19.2)	22 (22.4)			21 (20.8)	15 (21.4)		
Current smoker	21 (36.8)	33 (29.5)			17 (23.3)	38 (38.8)			32 (31.7)	23 (32.9)		
**Regeneration**												
Easy to take breaks			8.91 (4) [c]	0.063			9.01 (4) [c]	0.061			2.67 (4) [c]	0.615
Never	10 (17.5)	14 (12.5)			11 (15.1)	13 (13.3)			16 (15.8)	8 (11.4)		
Rarely	17 (29.8)	17 (15.2)			21 (28.8)	14 (14.3)			17 (16.8)	18 (25.7)		
Sometimes	15 (26.3)	28 (25.0)			20 (27.4)	24 (24.5)			28 (27.7)	16 (22.9)		
Often	10 (17.5)	37 (33.0)			16 (21.9)	31 (31.6)			27 (26.7)	20 (28.6)		
Always	5 (8.8)	16 (14.3)			5 (6.8)	16 (16.3)			13 (12.9)	8 (11.4)		
Sleep quality			0.94 [d]	0.865			1.94 [d]	0.637			2.12 [d]	0.591

Very poor	1 (1.8)	3 (2.7)	3 (4.1)	1 (1.0)	3 (3.0)	1 (1.4)
Poor	20 (35.1)	41 (36.6)	27 (37.0)	35 (35.7)	34 (33.7)	28 (40.0)
Good	35 (61.4)	63 (56.3)	41 (56.2)	58 (59.2)	59 (58.4)	40 (57.1)
Very good	1 (1.8)	5 (4.5)	2 (2.7)	4 (4.1)	5 (5.0)	1 (1.4)

Note. N = 171. [a] Two diverse participants were excluded from the sub-group analysis of sexes (n = 169) as a group of 2 cannot be statistically compared. [b] Normal weight: 18.5 kg/m^2 < BMI < 25 kg/m^2. Overweight: BMI ≥ 25 kg/m^2. No participants were underweight. [c] Chi-square test. [d] Fisher's Exact test. [e] Differing sample and sub-group sizes due to excluded participants; sex: n = 159, n_m = 52, n_f = 107; age: n_{age} = 161, $n_{<40}$ = 69, $n_{\geq 40}$ = 92; weight status: n_{weight} = 161, n_N = 97, n_O = 64. p < 0.05 for values indicated in bold.
